# Relationship based care – how general practice developed and why it is undermined within contemporary healthcare systems

**DOI:** 10.1080/02813432.2019.1639909

**Published:** 2019-07-12

**Authors:** Carl Edvard Rudebeck

**Affiliations:** Research Unit, Kalmar County Council, Kalmar, Sweden

**Keywords:** Generalism, specialisation, general practice, primary care, doctor-patient relationship

## Abstract

**Objective:** Investigating the state of generalism in medicine from the outlook of general practice.

**Line of argument:** General practice developed when its pioneers, in continuing relationships, learnt to know their patients through the variety of medical situations. From the 50s, there is an increasing literature on the virtues and challenges of relationship based general practice, and register-based research indicate its benefits. Generalist perspectives and person-centeredness are implemented in specialised care and medical education but need to be complemented by an input from relationship based general practice. The politically defined aim of primary care is not to balance the draw-backs of specialisation, but to provide medicine at the primary care level. In Sweden, and increasingly even in traditional strongholds of general practice, team-based primary care is thought to respond to increasing demands, filtering out non- and minor disease through triage, practicing task distribution, and moving the GP to a secondary level working with the ‘really sick’, in all a decline in direct contact between patient and GP.

**Conclusions: **When this happens, clinical medicine as a whole becomes drained of the practice of its human dimension. The lack of absolute proof of medical benefits cannot justify a disregard of the value of mutual knowledge and trust in the relationship, but still, in several countries, relationshipbased general practice will be hard to achieve for GPs planning their career. If the political winds should change, a sustaining profession of GPs preserving their relational ethos inside the team model, may be prepared to reform primary care.

**KEY POINTS**
- Proclaiming both biomedical breadth and the trustful relationship between doctor and patient, as a specialty, general practice embodies medical generalism.
- A direct input from the patient’s personal GP is necessary to make specialised care become more comprehensive and individualised.
- In reality, the team, practicing triage and task distribution, is increasingly replacing the doctor-patient relationship as working mode in primary care
- When the disease rather than the doctor-patient relationship, becomes the organising principle of primary care, medicine as a whole will be drained of the practice of its human dimension.

## Introduction

In parallel to specialisation in medicine, general practice has evolved from originally denoting the non-selected medical work outside hospitals to become the relationship-based generalistic medical discipline of primary care. Closely interrelated, the discipline and the organisation are still distinct. The size of the organisation does not necessarily mirror state of the discipline. In this paper I will try to catch the contents and features of general practice as the discipline has developed since the middle of the last century. Since generalism in medicine is more than general practice, I will also discuss the potential of existing comprehensive and person-centred approaches to cover the needs of generalist perspectives in specialised medicine. Raising doubts whether these approaches will do, my conclusion is that primary care organisations, built on relationship-based care, will be necessary to give medicine as a whole its necessary input of generalism. Then, analysing the vulnerable position of relationship based care in contemporary primary care organisations in Sweden and internationally, the article ends up in discussing the prospects of relationship based general practice, and the options for the future for GPs, both crucial for the profession to consider when forming its strategies for the decades to come.

## The emergence of general practice as a specialty

When the pioneers of general practice embarked on their mission, patient-centredness was not yet coined. Their competence was mainly about a breadth of diseases, but when they met and learnt from their patients in a non-bureaucratic fashion they also developed other drives and abilities beyond diagnosing and treating defined disease. Within the continuing relationship people stepped forward in their fullness of their lives, and through them also their families and the local context. Diseases had their consequences in everyday life, while strain and catastrophes were the soil of ill-health [1–3]. Symptom presentations were personal narratives [4]. The relationship was not merely a formal arrangement, but a personal obligation and an inexhaustible mutuality that brought about knowledge, trust, loyalty, and regard [5]. Growing through the career of the GP, the experience of many relationships taught the doctor about the universal vulnerability of body and soul, as well as about the cleverness and resilience that enabled the patients to cope with strain and medical challenges [6]. The GPs pricked up their ears when the symptom presentations signalled disease, but learnt by the same token the ways in which the biological logic expressed itself in bodily, spiritual, and social life. There was nothing more valid to fetch from the interaction than the GP’s own interpretations of the patient’s narrative and diagnostic considerations. This was always where diagnosing began [4]. In fact, the majority of ailments and symptoms never became reasons for encounter [7]. The patients consulted when their own experience had reached its limit, but they were themselves the first line and this was the precondition when appointing the GP to be their advisor. Therefore, the patients’ own capability of judging and handling their symptoms turned out to be an important ally [8]. From its very opening, the relationship was a joint venture of diagnosis and treatment. Within the continuing confirmation and renewal of the relationship, the GP then had the chance to feel increasingly at home and free to individualise the application of biomedicine in effective ways.

## The problems of the relation

The relationship competence was not solely about harvesting the advantages of the relationship, but also to face the challenges and problems that inevitably arise in encounters between doctor and patient. In this regard, urgent insights evolved quite early. When the psychiatrist Michael Balint in the 1950s in London, invited very hardworking GPs to discuss their difficult cases in seminars, the doctor’s role in the relationship became salient [9]. The doctor’s personality and non-reflected actions, captured by metaphors and concepts such as ‘the drug doctor', ‘the apostolic function', and ‘the dilution of responsibility’, sometimes contributed to maintaining the patient’s illness, or even made it worse. Since the running of the original seminars, GPs in many countries have found support in developing their understanding of patients by attending Balint groups [10].

Byrne & Long’s large study of tape-recorded consultations from the 1970s, revealed how easily rushed GPs forgot to invite patients to tell their view of the symptom, and why they had decided to consult [11]. The GPs’ biomedical reflex to attend to nothing but the eventual disease [4], a strong residue from the medical education and internships in hospitals, was a bias in relation to what the patients experienced and wanted to talk about. Within this tension the researchers unfolded the ‘patient’s agenda’, always crucial for the doctor to explore and a cornerstone of what we now describe as ‘patient-centredness’ [12].

The closeness of the GP-patient relationship was its strength, but also its hazard. Ian McWhinney, insisted that a GP has to strive for self-awareness to become a good doctor [13]. Acknowledging, and listening to, the patient, is not enough. To be a healer the doctor needs to strike the balance between involvement and detachment. Detachment is necessary to take responsibility, and to hold egocentric emotions back. Driven by over-involvement the doctor may, in fact, abandon the patient in crucial situations, and gradually drain him/herself of the inspiration that was to last for a whole career. McWhinney’s thinking became a powerful element in “The patient-centered clinical method” which he and his collaborators developed in the 1980s. To him, patient-centredness extended beyond being a technique for making patient conversations easier and more effective; it was a moral attention that made the deeper understanding of the patient a must.

## The breadth

The certain width and content of the competence maintained by the GPs of earlier days responded to the symptoms and conditions that made the patients want to see their doctor. Familiarity with the” common”, and vigilance against the serious and the rare, was the ethos of the first generation researchers in general practice when they registered and studied symptoms and diseases in their own practices [14,15]. Short but safe-enough pathways to diagnosis, or judgement of other kinds, applied in the wide and untamed symptom panorama, proved to be quite effective [16]. The symptoms displayed in a certain GP’s office did not just reflect the local epidemiology, but also the gross function of the health-care organisation, and within it, the role of primary care. Any direct access to other specialists would influence the demand. As many of the symptoms patients presented did not fit with those of the defined diseases, the symptoms attained an important role of their own in general practice [17]. The symptoms were a transition between the epidemiology and the individual patient, with the abstracted, disease specific experiences on the one hand, and a personal and often fugitive experience on the other. The challenge of interpreting them was as much about trying to understand the person and his or her experience as looking for the possible causes hiding in the body [6]. When no such cause was to be found, and the discomfort was significant, there was still a job to do to try to support the patient without getting trapped by the hazards described by Balint [9]. To some patients, mere living meant a lot of suffering. Their medical life-stories were also their fates [1,3] and the GP was their witness [2].

Rooted in practice, the generalism of general practice inevitably developed its double contents; the breadth of knowledge about diseases guided by the demand and adjusted to an appropriate level of responsibility, and the depth of the relationship with the patient. Every job description from the international bodies of general practice, every textbook and congress turned out to encompass these two dimensions [18]. In a few decades, general practice grew into an independent and global specialty. In the UK, the first textbook for GP trainees was published in 1972 [19]. National and international organisations for general practitioners were founded, schemes for vocational training were established, academic departments were built, and with them general practice entered basic medical education and became a research discipline.

## Specialisation and generalism

The big and highly specialised hospital offers both the breadth and depth of medicine, but to make the combination of breadth and depth come into its own, there is still a need for specialists that have an overview and are capable of making judgements beyond the limits of their narrow expertise, and who see the necessity to cooperate with other specialists and staff [20]. The emergency physician is the generalist of the acute stage of a disease or a trauma. In the USA, the former common model in which the family physician kept his or her overall responsibility even for the in-patient, is gradually substituted by the employment of hospital generalists – hospitalists [21]. In the Swedish discussion the suggestion has come up to integrate geriatrics, general medicine and general practice into an overall activity – ‘Generalistic medicine’ [22].

Only after listening to the patient’s personal account can the doctor make the interpretations and judgements that make person and medicine come together in constructive ways. Here, broader medical knowledge is not enough. That is why the ‘hospitalist’ model also has its critics even among hospital specialists, who believe that it, first and foremost, implies the involvement of still another doctor whom the patient does not know [23]. The strength of having one’s own GP at the bedside is more the trust underpinning conversations and decisions in critical situations. Looking world-wide, it is not common for GPs to accompany their patients into the hospital wards, and whether this would really work on a broad scale within a highly specialized hospital care is uncertain. There may yet be other and more flexible ways for the GP to help in adjusting clinical decisions to suit the individual patient, provided the will is there with all involved. Moreover, conversations along a working GP-patient relationship may prepare the patient, and his or her family, for situations of deterioration and hospitalisation. Strengthening relationship based general practice should therefore also lie in the immediate interest of specialised care. ‘Person-centred care’ [24] is a philosophy that has been developed in specialised medicine and it has much in common with ‘Patient-centred care’, so long central to the development of general practice [12]. Person-centred care has been demonstrated to have positive effects on the carer-patient relationship, outcomes of care, and on patient satisfaction [24]. From the outlook of scientific publications it seems that person-centred care has been taken up mainly by the nursing profession, and its impact on subspecialised doctors seems to be limited [25]. As regards medical education, several studies indicate – there are limitations in terms of conceptual clarity and measurability – a decline of empathy among students during medical school [26], but that are also results that suggest that such a development may be counteracted [27]. In basic medical education internationally, specific efforts are made to prepare students’ for taking on the doctor’s professional role in real life, including self-awareness, ethical understanding, and interacting in a patient-centred way [28,29]. Still, it is not probable that this reform will bring about a balance between specialisation and generalism in its humanistic sense in hospital medicine. Knowing the facts, and having the defined skills in response to the patient’s medical need will always have a very strong impact on doctors working in a primarily disease oriented setting, and this is reflected in the space given to human and relational aspects within the medical education as a whole. Improving the continuity of care within hospitals, and keeping the door open to the patient’s GP, need to complement the initiatives of generalism that come from within specialised medicine. The fact that patients on the whole seem to appreciate, or at least accept, the present state of affairs [30], reflects the intrinsically moral character of medicine; it is intended and expected to do good [31], but this does not warrant that the individual patient’s comprehensive biomedical and human needs are actually met.

## The development of primary care

As we have seen, medical generalism has a double connotation; breadth within the biomedical perspective, and a specific receptivity to the role of ill health in the patient’s life founded in the doctor-patient relationship. This is general practice, but rather than having the role of a specialty that by its characteristics balances the drawbacks of specialisation, its formal recognition is mainly attained by providing the point of first contact in healthcare. The organisational concept should however be primary care – a part of the total healthcare system with the politically defined aim to offer health care beyond the close reach of the hospitals, and to save the hospitals from the amount of medical problems that do not demand specialized resources. Prevention is also part of the commitment. The structure and role of primary care differs a lot among countries [32,33]. On the one hand, there are countries like UK, Norway, The Netherlands and Denmark that have national systems based on patient lists and gate-keeping, with the clearly defined task to provide the population with a first line of contact with health care. In these countries, the professional bodies of general practice have a certain influence on the planning of primary care. On the other hand, there are countries like France, Belgium and Germany that have less regulated systems, where patients may also see other specialists than GPs without referral. Swedish primary care has features of both; it is mainly public, and it is integrated into a national health politics with a strategic responsibility for prevention. Nevertheless, its concrete role in Swedish healthcare as a whole is unclear. In Sweden, the hospitals were dominant from early on, and so their share of all visits was great, and they were, and are still, attractive to both doctors and patients. It is significant that when the ‘National board of health and welfare’ developed its ‘Tenets for outpatient care’ (Principprogram för den öppna vården) in 1968, ‘outpatient care’ was the term employed [34]. Outpatient care was that which was left over when the hospitals had fulfilled their task. The only obvious place for the broad generalist was in remote areas of Sweden, where no specialised care whatsoever was available. In the cities, general practice would be limited within the triangle of general medicine, geriatrics, and social medicine, and in addition to the GPs, the health-centres would be manned by doctors from the major hospital specialties. General practice was defined conditionally as the spectrum of disease knowledge and skills needed at the medical coordinate where the individual GP happened to work. Although general practice later became a specialty of its own, The National board of Health and Welfare and the regional health authorities – the county councils – never changed this principal view. By an international comparison, the proportion of GPs of all doctors in Sweden has consequently lagged behind. The ‘Family-doctor reform’ from 1994, under a centre-right government, broke this tradition in introducing a national system of listing with a personal GP, but the reform got stuck within a year of launching it, when the social democrats returned to power[35]. The support from the county councils and the hospital specialists, and even from the GPs, never got strong and nor did the Swedish people back-up the reform strongly enough to force the decision makers to change their minds. Swedish society was simply not prepared for such a structure in its healthcare in contrast to what Norway achieved ten years later.

In order to withstand increasing demand, the commitment of most Swedish general practitioners has been considerably narrowed. The vast majority of health-centres have chosen to refer all first contacts to triaging nurses. GPs are expected to see the ‘really sick’ in a thinning zone between the conditions judged to demand specialised care, and the conditions handled by nurses, specialised on a lower level. In its radical form, the principle of task distribution states that any isolated problem or task, that for strictly medical reasons does not take a GP’s competence, could as well, or even preferably, be taken over by a nurse. As a mere knowing about a variable breadth of diseases, with the relationship with the patient as a background bonus, general practice becomes an artificial specialty, lacking any initiative of its own. Politicians and administrators, colleagues in the hospitals, and the structure of the staff in the health-centres decide its contents and development. Not so few GPs approve of this structure and accept the role as the doctor for the ‘really sick’ in primary care. Others run away from health-centres with a shortage of doctors to enrol with staffing agencies, choosing a future as mobile and locum doctors.

## Changing conditions even in strongholds of general practice

In primary care systems that are building on GPs being easily accessible, and known by their patients, it is quite obvious what general practice is about. A totally pragmatic definition of the specialty as “that which GPs do” has been substantial enough. The dual development of societies and health care now challenge general practice even in countries that formerly belonged to its strongholds. General features of all the western countries, the US included, are aging populations, with an increasing number of diagnosed conditions and medications [36], and increasing demands from healthy and anxious people [37–39]. This development is accompanied by financial constraints in primary care, a relative decrease of GPs or family physicians among all doctors [32] with an increased workload for the GPs and a decline in their accessibility. In the UK, these general features combined with a growth of bureaucracy and financial management in the midst of clinical work has pushed British general practice into a serious crisis, confirmed by the decline in the recruitment of young GPs [40,41]. In Norway, the margins of the list system – Fastlegeordningen – are being stretched [42]. Tasks are added, however without a decrease of the patient lists. Young doctors hesitate to take on the work-load and enough recruitment cannot be taken for granted. For many years, Denmark had a stable list system and an independent general practice profession that enjoyed considerable trust from the population. After a long-lasting conflict with the GPs’ national association, the responsible authorities – amtene – seized the legal right to add tasks to the contract with the GP’s without any financial compensation [43]. Although the work contents of English, Norwegian, and Danish GPs reflect the great trust given to them by politicians and administrators – differently from their Swedish colleagues they are not there primarily to do what their hospitals colleagues do not do – they still have to settle with what has been left over in terms of money. To rescue British primary care, and its general practitioners, the NHS has launched ‘General practice – forward view’, a reform that, besides increasing the number of positions for GPs, advocates triaging and task distribution, thus limiting continuity of care by a named GP [44]. A routinisation of monitoring chronic disease involving nurses is also a salient element in Dutch primary care [33]. Such a monitoring tends to ‘privilege the needs of the institution for data over the particular needs of individual patients’ [45,46]. A change of the GP’s role, from being above all a personal doctor, to become a leader of, and a consultant within, the health-centre team is gaining increasing support internationally from researchers, and also from the WHO [32,47]. The disease, rather than the relationship with the patient, shapes the organisation when general practice goes on the defensive. In the US, family physicians are also becoming increasingly scarce. ‘There’s little doubt that the front line of medicine — the traditional family or primary care doctor — has been under siege for years’. [48]. Patients with minor complaints or diseases now have rapidly growing access to “retail clinics”, usually run by big corporations and located where many people pass by, such as in pharmacies, grocery shops, and big-box stores. Without pre-booking, and with even smoother access than that provided by urgent-care clinics, patients are tended to by nurse practitioners or physician assistants. Some family physicians have responded to the challenge from the big companies by adding retail-clinics to their own offer. In distributing the work between other competencies than the doctor’s, the retail-clinics mimic the task-distribution of the team in European primary care, however often without the opportunity of a coordination and oversight by a designated physician. In a commercialised context, and with the breakdown of the doctor-patient continuity, quick remedies for isolated disease-episodes become an interesting market to a number of stakeholders. Another branch on the same tree is ‘Direct-To-Consumer Telehealth’. Both retail-clinics, and consultations on the net, have been demonstrated to increase the health-care spending by widening the scope of reasons for encounter [49,50]. To meet this growing demand within a generalistic primary care, rather than just pouring out more minute portions of care in an algorithmic manner, would take a substantial investment in family physicians/GPs, as well as a continuing generalist commitment to their patients even when their illnesses appear to be minor or trivial. This goes for the US as well as for Europe. Whether any of these conditions will ever have a chance to become satisfied, the present situation does not tell.

## The GP-patient relationship and outcomes, and the limitations of the outcome measures

Patients and GPs value relational continuity, and this is in itself a strong argument for providing relationship based care [51]. Also, there are many reasons to refrain from seeking one’s GP, and the person who has still found it necessary to do so ought to find the decision respected and accepted, rather than questioned through triage. The difficulty of proving in numbers that a good relational competence promotes good outcomes does not mean that it does not matter to patients whether they feel acknowledged or not [52]. In fact, if GPs were to assess the relationship solely in terms of medical outcomes, they would have degraded their involvement with the patient to mere tactics.

Still, from the perspective of health politics, positive outcomes would make the case even stronger. The data available are mainly observational or register-based, and the picture is not unequivocal, but with these reservations stated, there are many indications that people’s health benefits from having a personal doctor who is accessible, knows their situation, and who may help them find their way in the health-care system [53]. Looking at continuity across specialties, a recent study shows an interesting association between continuity and lower mortality [54]. As for the effects of relational continuity, it is hard to establish causal links between the ways doctors interact with patients and clinical outcomes [55]. A study in which several of the methodological challenges were taken into account, showed statistical associations between, on the one hand, the doctor being patient-centred, and on the other, improved health after two months, and fewer diagnostic tests and referrals [56]. The decisive factor was that the patient him/herself experienced that the communication was patient-centred, and especially that common ground was achieved.

Relational continuity has been shown to have statistical associations with a decreased demand for in- and outpatient hospital care [57]. Active listing and more consultations with the GP have been shown to be associated with decreasing mean days hospitalised [58], as has been shown for an intervention improving, among other things, the accessibility, continuity and comprehensiveness of care with the own GP [59]. A mere expansion of general practice within an inner city area, with formerly sparse primary care, was associated with a decrease in outpatient hospital services [60]. The balanced and adequate use of the total of resources in health-care is obviously a good thing. It counteracts medicalisation, an important task for personal GPs [61]. On the next level, the balance between the expenses for health-care, and for other major welfare projects in society, is obviously also a good thing.

But there may be adverse effects of listing combined with mandatory gatekeeping. Such systems have been shown to have a significantly lower 1-year cancer survival compared to systems without gatekeeping [62]. A harmful acceptance of long waits for initial investigations legitimated by gatekeeping is suggested to be the crucial factor behind the decreased survival. In a qualitative study from the same research unit, possible mechanisms of “doctor-induced patient delay” were identified [63]. The asymmetry, and a potentially impaired trust, imposed by gatekeeping may result in self-restrictive care-seeking, and concerns about causing unnecessary trouble to a well-known GP may also hold patients back. Although any causal relations between gatekeeping and reduced cancer survival are hypothetical, and also recently have been contested by contradicting findings [64,65], mandatory gatekeeping may inflict on the mutuality and diagnostic sensitivity of the doctor-patient interaction. The systems alone should however not be blamed for the possible drawbacks here discussed. GPs, to whom some of their patients do not care or dare to tell their most important concerns, have serious limitations of their relationship competence. Being a personal GP is a delicate responsibility that has to be recognised and trained, and diagnostic delays should motivate a scrutiny also of the relationship.

## The GP-patient relationship undermined within the healthcare systems

The results of the interactions in a GPs consulting room are not spectacular and convincing enough to make the political and democratic decisions about primary care deliberately favour the doctor-patient relationship. Hoping for more resources, the general practice profession refers to special cases, such as multi-morbidity and the programmatic prevention, as baits for the decision makers, while the potential key-role of the GP to offer a readiness to see and support persons with ill health of any kind, is gradually being sacrificed. What we now see, is how the Swedish model with big teams in large health-centres supported by an array of self-care and self-monitoring IT-applications, and with an inherent shortage of GPs, seems to become the model of choice even in countries with a formerly strong general practice [32]. The Relationship based general practice may be no more than a historical parenthesis, born when medicine was undeveloped, and finally becoming overrun by the specialisation and plain disease thinking. The experiences from the doctor-patient relationship made by GPs from all over the world, and once inspiring them to create a profession, are difficult to convey to others. Once the lead has been handed over to other than GPs, the doctor-patient relationship can hardly return as the organising principle for primary care. The fact that general practice prevails in its attribute as medical generalism in a broader sense, does not compensate for the lack of its concrete clinical organization, where the doctor-patient relationship is both the major interface between medicine and person, and the working mode. Without this naturally and constantly recurring linkage, the relationship competence will hardly develop from a common state of “courtesy” to a state of professional and informed “curiosity” [25], and consequently, general practice will lose the basis of its authority in healthcare.

The idea that the job of the primary care physician should be based on the relationship with the patient is also strongly questioned by the politically nurtured image of the informed, IT-competent, and empowered patient, negotiating with the doctor within a frame of hierarchical power [66]. Here, doctors, GPs included, are regarded to be the knowledgeable but exchangeable technicians, establishing a doctor – patient relationship that is mainly instrumental. What is forgotten then, a real risk, is how the division of responsibility actually has to be managed in real life: Who is to, or is capable to, decide about what, situation by situation? Lacking a personal GP to consult, patients’ position in deciding about their own health may actually be weakened [67]. Neither, is it probable that healthy people, having the access to large amounts of biological data about their own bodies will decrease their need to consult GPs or other specialists [68]. Rather, there will be a demand for having the data professionally deciphered. The continuing development of IT will, beside its benefits, increase such problems where technology wrongly is believed to outdo the doctor-patient relationship and other relationships. This kind of misunderstanding in society will seriously undermine a realistic commitment of general practice, even more so if technology is given financial priority over GP manpower.

## The GP’s options

Although the conditions for maintaining or developing relationship based general practice differ a lot among countries, the critical traits discussed in this paper belong to a development of society and healthcare that may be discerned internationally. Young doctors who aim for general practice, should not take a relationship-based practice for granted, although this is the ideal that drives many of them. So what are the options for the individual practitioner?

On closer examination, general practice is not one, distinct profession, if one by ‘profession’ presumes an explicit and coherent knowledge-base and practice. The developmental stages of general practice, disease generalism, relationship-based practice, and primary care medicine all live in parallel also as differing understandings of what GPs’ competence basically is about, and of how their work should be organised. Depending on which of the three emphases the individual GP identifies with, the present squeeze of general practice has differing implications. Many “relationists” will see serious obstacles to obtaining reasonable standards of care, stretching themselves beyond their personal limits to be accessible and supportive for their patients. When overwork has been going on long enough they retire prematurely, lessen their work-hours or change specialty, which makes the situation even tougher for those who are left [40]. Therefore, before deciding on a definite career, the young doctor, should really consider, and try in reality, how close to the ideals she or he needs to be to see the job as worth-while. Indeed, there are relationists who seem to maintain their inspiration from and for the relationship whatever the conditions for developing it. They are the resilient relationists, preserving the tradition of general practice within primary care organisations that are, in fact, deaf to this tradition. However, they do not have the number and strength to fill the role of a full-fledged profession.

The future seems to belong to primary care medicine, where the GP is the doctor of the disease and the organisation rather than of the patient and the relationship. Many GPs who find themselves in this role would basically be relationists who have seen no alternatives. They probably try to make the best out of the situation, with more or less success, and thus with more or less frustration [40]. The critical transition from relationship based practice to primary care medicine is when the relationship is no longer recognised as a vital professional asset. As long as the doctor-patient relationship is valued and given priority by the whole staff, dividing the job according to situation and what benefits the patient, will be no problem.

The devoted disease generalists, who regard the disease competence to be the real competence and take the patient relationship for granted but without considering that it takes and develops a professionally crucial competence, would experience much of inner-city primary care medicine as too limited and bureaucratic. For young doctors, with a primary and broad interest in the biology of the human body, it should be more attractive to practise far away from hospitals, and/or to take on emergency medicine. If faithful to the list system, many of the remote area GPs will turn into the explicit or implicit relationists, like the pioneers of general practice once were educated by the reality of their practice.

Everything that is good is good. A good consultation is never in vain, but there are strong indications that relationship based general practice, in many countries, will stay but an ideal in a harsh world of forgetfulness and far-reaching compromises. The large scale and bureaucratic primary care organisations prevent relationship based general practice from being realized [69], and even more from being the organising principle of primary care. The perspectives of primary and secondary care will become rather much the same; not a catastrophe for a nation, but a great inadequacy for its citizens when ill, as well as for the working and financing of its total healthcare. Seen as a whole, clinical medicine needs the practice of its human dimension.

The future of relationship based general practice will then be the sum of individual practitioners who try to live up to its ideals and standards, within organisations that mostly have other agendas, or on islands of independence, which these GPs themselves manage to create. Very much will depend on their drive to build and sustain relationships with their patients, and on their ingenuity in dealing with severe limitations and in establishing informal and working cooperation with GPs who share their values. The associations and academic departments of general practice, must engage in this process, consistently embracing the discipline in its defining complementarity of biomedical breadth and relational depth. If, by chance, societies later on rediscover the need for continuous patient-doctor relationships in primary care available for all citizens, the will and competence to lead a sustaining profession in reorganising relationship based care, and in training young GPs for their role in the healthcare systems, will be in place.
